# Hybrid Governance: Negotiating justice for health services in informal urban settlements in Dhaka, Bangladesh

**DOI:** 10.1093/heapol/czaf081

**Published:** 2026-06-29

**Authors:** Bachera Aktar, Kim Ozano, Sabina Faiz Rashid, Linda Waldman, Sally Theobald

**Affiliations:** Department of International Public Health, Liverpool School of Tropical Medicine, Pembroke Place, Liverpool, L3 5QA, United Kingdom; BRAC James P Grant School of Public Health, BRAC University, Floor 10-13, BRAC Tower, 65 Mohakhali, Bir Uttam AK Khandakar Road, Dhaka 1212, Bangladesh; The Stop, Collaboration and Listen (SCL) Agency, Five Fords Gate, Bridge Road, Wrexham, Wales, LL13 9PS, United Kingdom; BRAC James P Grant School of Public Health, BRAC University, Floor 10-13, BRAC Tower, 65 Mohakhali, Bir Uttam AK Khandakar Road, Dhaka 1212, Bangladesh; Institute of Development Studies, Library Road, Brighton, BN1 9RE, United Kingdom; Department of International Public Health, Liverpool School of Tropical Medicine, Pembroke Place, Liverpool, L3 5QA, United Kingdom

**Keywords:** hybrid governance, informal urban settlements, urban slums, health governance, Bangladesh

## Abstract

This paper examines the complex governance landscape of public health service delivery in the informal urban settlements of Dhaka, Bangladesh, through the application of Hybrid Governance and Urban Political Ecology (UPE) frameworks. Rapid urbanization and insufficient state capacity have led to the development of informal settlements, where conventional governance structures are absent. In this context, hybrid governance systems emerge, which are characterized by collaborations and conflicts among formal authorities, non-state and informal actors, and local community members. In-depth empirical data were drawn from multi-method qualitative research applying community-based participatory research (CBPR) approaches, conducted between 2021 and 2023, in two informal urban settlements in Dhaka, Bangladesh. This paper introduces a “Multi-layered Hybrid Governance” (MHG) framework that explains the complex governance arrangements of public health service delivery in informal urban settlements. The MHG framework features the fragile yet resilient nature of collaborations between formal authorities (state-level governing bodies), formal governance actors (elected local government representatives) and their informal representatives (community leaders, local elites), private service providers (including NGOs), and community members, revealing how public health services are negotiated and legitimized within these informal contexts. The findings suggest that hybrid governance is a form of community resilience that can enhance service delivery in the absence of effective state intervention. However, it can also reinforce existing inequalities and introduce new challenges to residents, especially to those without political connection or strong social networks. This paper urges policymakers and researchers to look beyond conventional health governance models and recognize the limitations of the existing systems. By presenting the MHG framework as an analytical tool for learning health systems from the perspectives of those on the margins, we advocate for more inclusive, resilient governance approaches for promoting justice and ensuring sustainability within fragmented urban health systems.

Key messages

- The Multi-layered Hybrid Governance (MHG) framework presented here features the adaptive, resilient, and relational nature of hybrid governance, which evolves through flexible, negotiated relationships between formal authorities, NGOs, informal community leaders, and residents.
- The MHG framework enables analysis of how resilience-oriented governance enables access to public health services while reinforcing inequities, affecting who gets access to services.
- The MHG framework recognizes the embedded power of informal community actors, such as community leaders, operating outside institutional frameworks, yet significantly influencing service delivery and access in informal urban settlements.
- The MHG framework offers an analytical tool for policymakers and practitioners for learning health systems from the perspectives of those on the margins to develop equitable and sustainable health service delivery models within fragmented urban health systems.

## Introduction

In the Global South, rapid unplanned urbanization has led to complex governance challenges, especially in areas where state governance is weak, and informality has become a key characteristic of governance. In cities like Dhaka, the capital of Bangladesh, the rapid population growth has far surpassed the capacity of the existing urban infrastructure. As a result, informal settlements have emerged as a dominant form of urban habitation for poor city residents (Devas et al. 2004, te Lintelo et al. 2018). About 1 billion people worldwide reside in informal settlements, which is projected to double by 2030 (UN-Habitat 2022). This highlights the urgent need to address the resulting health inequities in these settlements (Ezeh et al. 2017).

National policies, local power structures and politics, and interactions among state, non-state, and informal community actors are fundamental in shaping the governance ecosystems of informal urban settlements (McCann 2017). The exclusion of informal settlements from national health planning and urban development raises critical questions about health systems’ justice, as residents in these areas typically face worse health outcomes and have lower access to essential services compared to other urban populations (Corburn and Sverdlik 2017, Elsey et al. 2019). Limited reflexive practices within health systems’ learning (Sheikh and Abimbola 2021) and neglect of less visible governance actors and processes within growing informal health settings have deepened inequalities.

The limited reach of states into these informal settlements has given rise to hybrid governance systems, where formal authorities, state, non-state, and informal actors interact in overlapping, negotiated, and often contested ways (Hackenbroch and Hossain 2012, Hossain 2013, Jones 2017). This paper introduces the Multi-layered Hybrid Governance (MHG) framework as an analytical tool to assess governance of health service delivery and analyze how power, politics, and institutional ambiguity influence access to health services in urban informal settlements in Dhaka, Bangladesh. The MHG framework helps to analyze hybrid governance as a resilience-oriented governance system that addresses immediate needs by bypassing or collaborating with formal systems.

### Political and policy context of urban Bangladesh

While the Bangladesh government has made progress in national-level health indicators, it continues to face persistent challenges in its health system. Despite rapid urbanization in Bangladesh, urban health governance remains fragmented and exacerbated by institutional ambiguities between the Ministry of Health and Family Welfare and the Ministry of Local Government (Adams et al. 2015). The absence of comprehensive urban policies, inadequate resource allocation, and unclear jurisdictional responsibilities of local government bodies [e.g. city corporations and Ward Councilors (WCs)] result in inequities in public service provision, including health services in informal urban settlements (Banks 2008, Afsana and Wahid 2013, Adams et al. 2015).

Informal urban settlements now host over 40% of Dhaka’s population (BBS 2023). These settlements are characterized by high population density, substandard housing and living conditions, and poor-quality water and sanitation services, creating greater health challenges for the residents (World Bank 2007). Structural violence in these settlements is enacted through physical evictions, systematic denial of legal recognition (World Bank 2007, Hossain 2013), and lack of state-provided basic services, such as water, sanitation, and healthcare (World Bank 2007, Hossain 2013). This makes it critical to understand the informal arrangements that have emerged in this policy vacuum and their impact on justice and sustainability in health systems.

The health needs of diverse urban poor, particularly those residing in informal settlements, have been systematically neglected (Afsana and Wahid 2013). This neglect is rooted in policy inaction and the complex governance ecologies that define service delivery in these areas (Arise Consortium 2024). As a result, these informal urban spaces have developed their own forms of hybrid governance to support sustainability for some, although not for all residents, and in doing so, undermine both justice and sustainability (Arise Consortium 2024).

Bangladesh’s current political and policy context (post-July 2024 with an interim government) provides a timely backdrop for examining hybrid governance to ensure improved health, justice, and urban sustainability for poor residents of informal settlements in the future. The interim government, led by Chief Adviser Dr Muhammad Yunus, initiated several reform commissions, including the Health Sector Reform Commission, established in November 2024. The Health Reform Commission emphasized on decentralization, community engagement, and strengthening primary healthcare and recommended an allocation of 15% of the national budget for the health sector (The Daily Star 13 May 2025, The New Age 22 May 2025, The Prothom Alo 6 May 2025). However, to implement the reforms, it is important to recognize the reality of the urban governance ecosystem at national and local levels, which may compromise justice in health systems by reinforcing exclusion and inequity. In response to these challenges, this paper offers grounded, empirical evidence of health service governance in informal settlements in Dhaka, Bangladesh.

### Hybrid governance in informal urban settlements

Governance in informal settlements is non-linear and functions through multidimensional informal–formal interactions mediated by political alliances and local norms (Paller 2015, Goodfellow 2020, Arise Consortium 2024). The concept of hybrid governance helps to capture this complexity. The hybrid governance system operates through the informal blending of formal and informal systems and acknowledges the growing role of diverse non-state and informal actors in decision-making and implementation (Boege et al. 2009a, Mcdermott et al. 2015). These arrangements often involve collaboration, negotiation, and power-sharing between these actors.

Hybrid governance, as a concept, emerged from the critiques of state-led formal governance models, which often lack the capacity and resources to address complex societal challenges (Anciano and Lombard 2024). It draws on governance theories that emphasize the importance of multi-actor involvement in collaborative and network governance (Boege et al. 2009b, Mcdermott et al. 2015). Hybrid governance emerged as short-term strategies for seeking justice in accessing urban health services. In the context of health policy and systems research (HPSR), hybrid governance offers both a theoretical framework and a practical approach to learn about health systems and resilient governance.

In the rapidly growing informal urban spaces, governance encompasses formal policies and institutions as well as informal rules, power brokers, and spatial claims. Informal settlements operate within a gray space of legality, informality, and negotiated survival (Yiftachel 2009, Arise Consortium 2024), where hybrid governance becomes both a coping mechanism and control system. This supports the notion that hybrid governance “fills the gap” where the state is weak or absent (Boege et al. 2009b). In Bangladesh’s pluralistic health system, hybrid arrangements act as informal workarounds to systemic exclusion, especially for the poor, politically disconnected, or socially marginalized people (Hossain 2013, Banks et al. 2020).

In these settlements, hybrid governance systems determine not only who gets access to health services but also how services are delivered, to whom, and under what terms. Politically powerful and relatively well-off people play a critical intermediary gatekeeping role in this hybrid system by establishing a patronage relationship with political leaders and elected local government representatives, such as WCs (Hackenbroch and Hossain 2012, Hossain 2012, 2013). Hossain (2013) noted that a dynamic “mutual support system” exists in informal urban settlements in Dhaka city, where political leaders utilize community leaders for mobilizing votes and, in return, give them political power and control over utility supply businesses such as water and electricity. These patronage relationships remain fluid and result in a disproportionate distribution of resources (Hackenbroch and Hossain 2012, Hossain 2013).

However, most existing hybrid governance theories and frameworks describe hybridity as a stable coexistence of state and non-state authorities, which simplifies the complex and multi-level governance realities in urban areas into the dichotomy of “formal (state)” and “informal (non-state)” (Boege et al. 2009b, Meagher 2011, Mcdermott et al. 2015). One limitation of the existing hybrid governance literature is its insufficient analysis of how formal and informal governance actors and mechanisms coexist, collaborate, or compete within informal urban settlements of the Global South, such as Bangladesh, which directly influence health governance in these communities (Yasmin et al. 2022, Abdulhadi et al. 2024, Kaur et al. 2025). In those informal contexts, governance is negotiated through everyday practices, informal norms, and local power structures at different levels, which Hackenbroch and Hossain (2012) referred to as “hybrid institutional arrangements”.

To analyze the complex hybrid governance systems in informal urban settlements, we draw on the urban political ecology (UPE) framework. UPE emerged in the 1990s, drawing from political ecology, urban studies, and social theories to address the social dimensions of environmental problems in cities (Swyngedouw and Heynen 2003, Cornea 2019). Theoretically, UPE is rooted in critical geography and Marxist political economy, considering the city as a socio-ecological assemblage shaped by power relations, which produces unjust and uneven urban landscapes (Swyngedouw and Heynen 2003). It highlights how power dynamics, social inequalities, and capitalist development shape urban environment (Rice 2014, Parizeau 2015, Cornea et al. 2017, March and Swyngedouw 2022). The UPE framework offers insight into how justice, sustainability, and resilience intersect in urban spaces, shaping living and governing environments (Heynen et al. 2006). It analyzes service gaps as politically produced vulnerabilities that disproportionately affect the residents. The UPE framework offers valuable insights into HPSR, informing the development of more equitable, just, and sustainable urban health policies and interventions. Drawing on the UPE framework, this paper critically examines urban health governance in informal settlements, considering three case studies within Dhaka city and situating this within the broader discourse of HPSR. While hybrid governance is an emerging topic in development literature, few studies offer empirical and grounded frameworks to guide HPSR. There is a pressing need for HPSR to shift from disease-focused interventions to inclusive, governance-oriented research that actively engages communities, thereby learning about and holistically strengthening urban health systems. The primary aim of this paper is to explore and explain how hybrid governance structures influence public health service delivery in informal urban settlements in Dhaka, Bangladesh.

**Figure 1.**
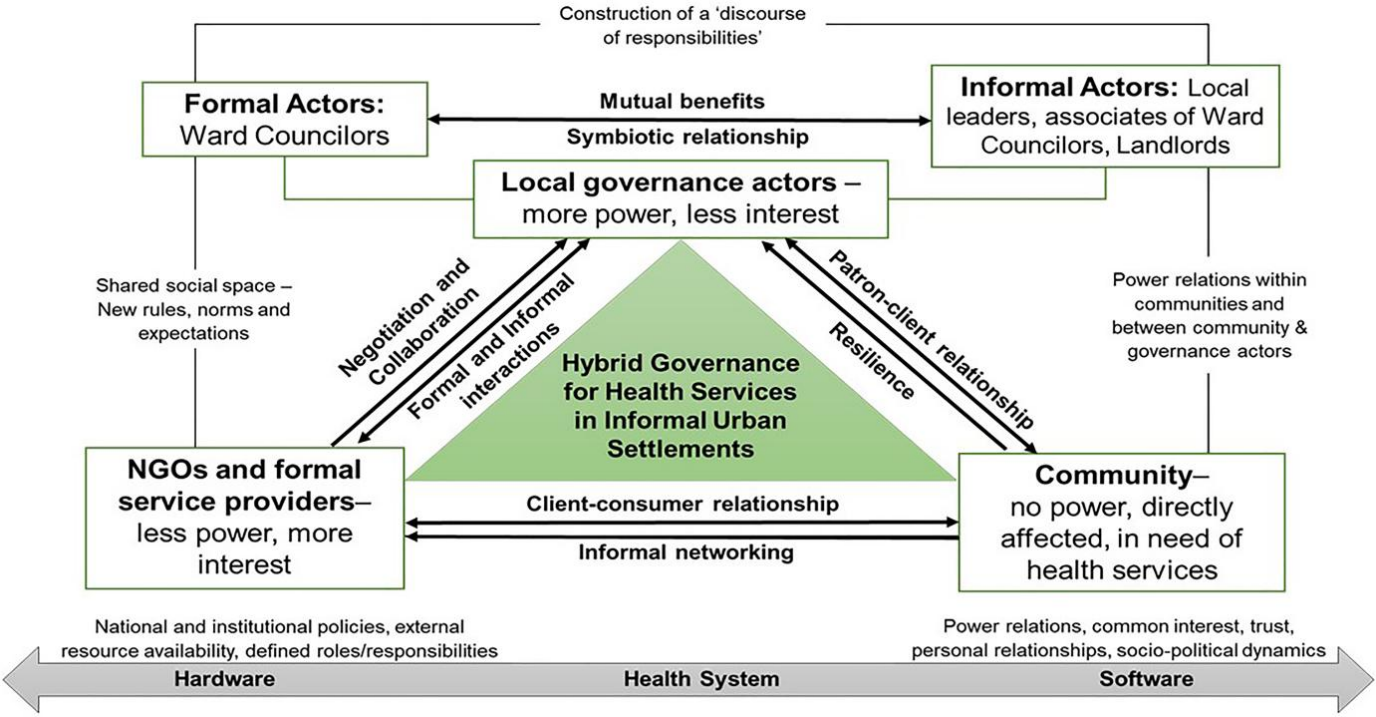
MHG for health services in informal urban settlements.

This research provides evidence on how structural, social, and political forces influence health service access and perpetuate injustice. We developed a “Multi-layered Hybrid Governance” (MHG) framework to illustrate the fluid positionality of actors in informal settlements across different layers of informal governance, where community leaders serve as informal gatekeepers, NGOs function as intermediaries, and residents oscillate between being clients and citizens depending on the context. The governance layers represent the local power hierarchy, and actors’ roles and influence vary according to their hierarchical positions (see Fig. 1). Our framework also incorporates an intersectional analysis to investigate how the socioeconomic status and political affiliations of residents in informal urban settlements influence their access to public health services.

Through this paper, we provide a more nuanced understanding of governance in complex urban settings, thereby contributing to a deeper understanding of health systems, both within Bangladesh and globally. This will also inform the ongoing health sector reforms in Bangladesh, aiming to improve justice and sustainability in urban health systems for residents of informal settlements.

## Methods

### Study settings

This study explored health governance in two informal settlements in Dhaka city, designated as Site A and Site B in this paper, to protect participants’ confidentiality due to the politically sensitive information gathered.

Site A, situated on land owned by a government agency within the Dhaka North City Corporation (DNCC), is one of the city’s oldest and largest informal settlements. Many long-term residents, who had lived here since its establishment, were local landlords, and many of them gradually became local leaders or influential figures within the community. This settlement has a history of frequent evictions by the state, which is not unusual, as settlements have faced demolition and eviction over the years. More than 22 NGOs provided various services, including water and sanitation, health, and education, during the data collection period.

In contrast, Site B is a comparatively new and small settlement situated on a mix of privately owned and Bangladesh Railway’s vacant land on the outskirts of Dhaka South City Corporation. The area features scattered housing integrated with surrounding multi-story buildings. The population largely comprises workers from nearby factories. Better-off residents own most of the surrounding multi-storied buildings, some of whom have built makeshift wood and corrugated iron houses along the railway lines, which they rent to tenants. This part of Site B frequently faces State-issued eviction notices. Site B had fewer NGOs and service providers than Site A, which may be due to its geographical location, smaller size, and lower political visibility.

These settlements, like others in Bangladesh, lacked access to government services. There was one NGO health clinic located near each site. The Site A clinic was operated under the Urban Primary Healthcare Service Development Programme of the Ministry of Local Government, and the Site B clinic was a pilot collaborative initiative of the Ministry of Health and Family Welfare. There were many private hospitals surrounding both settlements. However, poor residents mostly sought health services from local unregistered pharmacies and distant public hospitals.

In Bangladesh, the politics of informal settlements are shaped by land value, political patronage, and strategic utility, leading to unequal treatment among different types of settlements. Those with political patronage or strategic electoral importance often receive informal protection and may access basic amenities despite lacking formal recognition (Banks 2008, Banks et al. 2011). Because of this uneven power and resource distributions, informality becomes the norm of governing urban space, where the rights of the most marginalized are systematically neglected (Roy 2005).

### Data collection

This paper presents data from a multi-method (Creswell and Creswell 2018) qualitative research conducted in two informal settlements in Dhaka between December 2021 and February 2023. Data were collected through multiple participatory qualitative methods, including 10 governance network mapping (GNM) exercises (group sessions with a duration of 60–120 min) with 68 participants using the Net-Map tool (Schiffer 2007), and governance diaries (GD) method (Loureiro et al. 2020) through repeated in-depth interviews (two to three visits over three months, with an average duration of 90 min/interview) with 16 families. We recruited two community members as co-researchers (Co-Rs) from each study site. As residents of the same community, the Co-Rs shared critical insights of their communities that other methods could not capture and helped validate and interpret data collected. They also assisted in organizing and facilitating GNM sessions and GD interviews. We obtained informed written consent from all participants prior to conducting GMN sessions and GD interviews. We conducted all sessions and interviews in Bangla, the native language and audio-recorded them. Table 1 presents a brief profile of the study participants.

**Table 1.** Profile of study participants

Characteristics	Site A	Site B
A. Governance network mapping (Net-Map) sessions		
Total sessions	5	5
Total participants	37	31
Female	22	22
Male	15	9
Types of participants		
Community-based committee members	10	8
NGO frontline workers	6	2
Persons with disabilities	6	9
Informal sector workers	15	12
Age range (in years)	18–68	24–75
Duration of staying in the settlement (in years)	18–40	5–52
B. Governance diaries—repeated in-depth interviews		
Participating families	9	9
Primary participants’ age range (in years)	25–50	35–75
Primary participants’ gender		
Female	6	8
Male	4	1
Transgender		1
Duration of residency in the settlements (in years)	5–30	3–15
Types of participants		
Community leaders	3	2
Community-based committee members	1	1
Daily wage earners	2	4
NGO workers	2	
Traditional birth attendant		1

### Data analysis

Two trained transcribers transcribed audio records in Bangla, which the lead author (B.A.) subsequently quality-checked. Using NVivo 12 software, a combination of deductive and inductive coding approaches was applied, which allowed both theory-driven analysis and the exploration of emergent themes within the data (Bingham 2023). B.A. performed thematic framework analysis (Goldsmith 2021), applying the MHG framework (Fig. 1).

## Results and discussions

We developed the MHG framework based on the empirical data from this research to explain the complex and evolving nature of health governance in informal urban settlements. We first explain the MHG framework, flowed by three examples of hybrid governance arrangements for accessing public health services in these settlements. Finally, the drivers and challenges of hybrid governance are discussed within the broader discourse of UPE, community resilience, health systems justice, and sustainability.

### Evolution of multi-layered hybrid governance in informal urban settlements

Our findings revealed that health services in informal urban settlements function through a highly context-specific, MHG arrangement—a complex informal public-NGO-community collaboration system. Figure 1 illustrates the MHG framework, followed by an explanation of this framework under four subthemes: (i) Governance layers and actors’ positionality; (ii) Interactions and relationships between actors; (iii) Shared social space and networked governance; and (iv) Hardware, software, and trust in governance.

#### Governance layers and actor positionality

As depicted in Fig. 1, the hybrid governance system has three broad categories of actors: governance actors, service providers, and community members. The positions of different actors at different levels are flexible and change at different times based on their roles and the purposes of their actions.

Formal governance actors (WCs in this framework) and their representatives (community leaders, local elites) are “local governance actors” at the top tier (macro level) of the hybrid governance network. These local governance actors utilize the decision-making power gained through their positions within the local government structure (WCs) or their political affiliations (WCs’ representatives). These local governance actors possess an intimate understanding of local dynamics, granting them a substantial influence within the community, including the ability to influence service delivery at the community level. For example, one male NGO worker stated:

If you do anything without communicating with them [referring to local leaders], you cannot do it properly. If you inform and involve them, you can complete your task easily and effectively. (Male NGO Worker, Site A, GNM)

Personal, family, and political connections influence the overall leadership of the informal governance network, as shown in the illustrative quotation below.

A few families control our slum. Power and leadership carry forward from generation to generation. Only the children of the “sarders” [leaders] can be a sarder. (Female Youth Volunteer, Site A, GNM)

However, informal leadership within these settlements is fluid and changes in response to the prevailing national political landscape. A notable instance occurred in Bangladesh following the political shift in August 2024, which reshaped the leadership structure in Sites A and B. The previous regime’s 15-year tenure fostered a continuity of leadership tied to generational family connections. However, the national-level power transitions resulted in a redistribution of influence among local governance actors, including the elected local government representatives—the WCs. Families associated with the outgoing government lost their political authority and some were forced to flee or hide. The political appointments of WCs were also annulled following the dismissal of the previous government. Individuals affiliated with political parties that the previous regime had oppressed emerged as new leaders. This phenomenon illustrates the precarious nature of leadership in these informal governance networks and highlights the profound impact political transitions have on community dynamics and local governance.

Formal service providers, such as NGOs and other private providers, are situated at the meso level in the hybrid governance structure. As the primary health service providers in informal settlements, NGOs play a crucial intermediary role in connecting formal and informal actors, particularly by establishing community-based committees (CBCs), which also act as their gateway to the community. However, NGOs have less power within the hybrid governance structure because of their informal dependency on the local governance actors for access to the community. Other formal service providers, for example, the private sector, have little to no influence on community decision-making, as the respective communities considered their approach to be commercial and market-oriented despite using their services.

Individual community members are positioned at the micro level of the hybrid governance structure. However, some may transition to the meso level by joining CBCs or building strategic alliances with those in power (WCs and their representatives), or macro level by joining politics.

#### Interactions and relationships between actors

Hybrid governance is an emergent outcome of interactions between various actors at different levels, both internally and with external stakeholders, to solve health problems and access health service delivery (Kickbusch and Gleicher 2012). Hybridity is not static; it is highly context-specific, politically constructed, and evolves through informal negotiations and strategic alliances between formal and informal actors. These relationships are characterized more by their political nature and mutual reciprocity than by legality or formal accountability. The complex interactions among these actors are significantly influenced by social relations, local power dynamics, shared interests, and collaborative advantages (Boege et al. 2009a, Banks et al. 2011, Hossain 2013, Banks et al. 2020). These social networks and interactions shape governance structures that often blend formal and informal practices (Freire and Stren 2001).

In the MHG framework (see Fig. 1), we describe the patron–client relationship as a “symbiotic relationship of mutual benefits”. The illustrative quotations below describe how resources are distributed in Site A through patron–client relationships.

Any distribution of resources or aid in the area requires permission from the local Ward Commissioner. Once permission is granted, the actual distribution is handled by selected individuals who work as his representatives. ……. There are 2–3 such leaders here. Each, and everything comes through them. (Male, person with disability, Site A, GNM)

The councilor distributed food ration cards during COVID. Those who worked for him received those. I got one as I did an election campaign for him. (Female, community leader, Site B, GD)

In this context, informal governance actors, such as community leaders and local elites, serve as WCs’ representatives and control access to resources and influence service delivery by exercising their power. In this hybrid arrangement, community members access services not through formal institutional channels, but rather through patron–client networks that involve WCs, NGOs, and community leaders.

#### Shared social space and networked governance

The hybrid governance system often cultivates informal governance networks that are self-organized, self-governed and politically driven. They operate under unique informal rules and policies, contributing to a distinct urban ecological context (Swyngedouw 2005). A key characteristic of hybrid governance is “shared social space”, where formal and informal actors coexist and cooperate, often without clear rules, and sometimes foster the evolution of new rules and norms. These shared spaces are maintained through mutual dependence, constituting an informal “discourse of responsibilities”. For example, NGOs and formal service providers collaborate with formal and informal governance actors, maintaining both formal and informal interactions, and often negotiate with these governance actors to access settlements, gain local trust, and deliver services. The following statement by an NGO field worker illustrates the informal rules for NGOs operating in informal settlements.

We must communicate with WCs, local elites and community leaders who have lived in this area for many years and look after local issues. We cannot do any work without communicating and interacting with them. Once we establish this connection, we do not face problems working with them. (Female NGO Field Worker, Site A, GNM)

Local governance actors rely on NGOs for resources and services and utilize their connections to channel benefits to their constituencies within the communities, thereby strengthening their own social capital. This aligns with UPE perspectives, where urban services are governed by fluid, negotiated ecologies of power and access (Freire and Stren 2001, Swyngedouw 2005). The interplay between formal and informal governance arrangements further emphasizes the importance of context in shaping health and social outcomes in informal urban settlements (Lemos and Agrawal 2006, Brondizio et al. 2009).

#### Hardware, software, and trust in governance

The MHG framework integrates both the “hardware” (infrastructure, finance, etc.) and “software” (relationships, trust, norms, etc.) of health systems (Sheikh et al. 2011). The intersections between these components influence health service delivery and access in informal settlements. Trust is not a prerequisite for collaboration in hybrid arrangements. Collaborations happen through transactional relationships driven by material benefits. This challenges the conventional view of trust as a fundamental component in governance networks. “Instrumental trust” or strategic dependency based on pragmatic collaborations (Gilson 2003) may be more relevant in hybrid governance in informal contexts. However, “instrumental trust” can lead to fragile collaborations if actors’ motivations and interests change, which is common in hybrid arrangements.

### Examples of hybrid governance for health services

This section presents three examples of “hybrid governance arrangements” for health services in informal urban settlements. By critically examining these arrangements, we aim to contribute to the discussion on just health systems, emphasizing the importance of adaptive governance structures as a means of fostering community resilience to meet informal settlement residents’ health needs.

#### Example 1: Hybrid governance in practice—Formalizing water access in Site A

**Figure 2.**
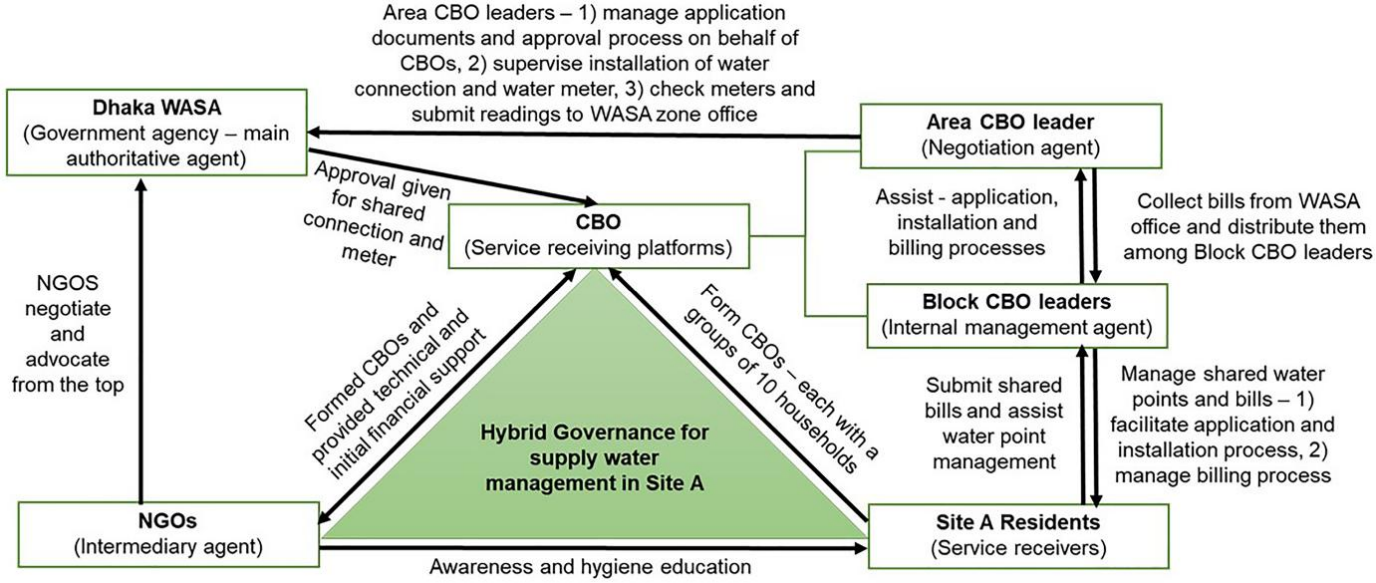
MHG of supply water management in Site A.

The provision of legal, piped water from the Dhaka Water Supply and Sewerage Authority (Dhaka WASA) in Site A serves as a strong example of hybrid governance in practice. This example illustrates an effective collaboration between a government agency, the communities, and NGO actors in delivering a sustainable solution to address systemic service delivery failures, breaking the syndicate of informal vendors and political brokers who previously controlled the water supply. Like many other informal settlements, Site A residents relied on informal water vendors who operated without regulation and exploited residents by charging high fees for poor-quality water with associated health risks and causing financial strain. The water access problem was compounded by a lack of recognition of this as a problem from the Dhaka WASA, as informal settlements were considered “illegal” and thus ineligible for formal service provision. This exploitative situation prompted collective action. Figure 2 summarizes the overall process of securing a legal water supply connection from Dhaka WASA and the internal management system.

The fight for a legal water connection in Site A, a basic necessity and civic right for city residents, began with support from two NGOs—WaterAid and DSK—known for improving water and sanitation in low-income and informal settlements. In 2005, a Community-Based Organization (CBO) was formed and registered as a federation, also supported by WaterAid and DSK, to advocate for formal water connections from Dhaka WASA. This CBO served as the liaison between residents and Dhaka WASA, leading the advocacy campaign. The NGOs provided technical assistance, initial funding, and acted as intermediaries between the community, Dhaka WASA, and the DNCC. The CBO negotiated with Dhaka WASA to secure legal water connections for slum residents. Persistent lobbying, not just from Site A’s CBO, but from 326 CBOs in Bangladesh’s cities, catalyzed broader institutional and policy reforms, leading to the creation of a Low-Income Community (LIC) Wing within WASA in 2010. This institutionalized a community-led water management mechanism for extending services to urban poor settlements by amending the policy that previously required a holding number to be eligible for a legal water connection. Through persistent negotiations and strategic alliances, the CBO successfully secured a 5-year formal agreement with WASA in 2011, resulting in the installation of over 240 legal water connections at Site A by 2022.

Since 2011, we’ve been receiving piped water through formal agreements with WASA. We now have over 240 legal water lines in Site A alone. People no longer wait in line for hours; water reaches their homes. This is dignity. (Male Community Leader, Site A, GD)

Site A residents are required to submit paperwork and a deposit to Dhaka WASA through a designated CBO leader in order to access this water supply. After payment and approval, WASA install a metered water line connected from the main zonal line to a local water reservoir designated for the specific CBO. Some residents paid a deposit of BDT10,000 (approximately £62) to the CBO leader for the connection, while others incurred no deposit but paid a local plumber, hired by the CBO leader, for the installation. Some households received individual meters, while others shared a meter among ten households.

CBO leaders managed the water supply lines, which included reading the meters, submitting these readings to the zone office, receiving bills, and distributing them to users. Users pay their monthly bills directly to WASA based on their water consumption. Individual users receive their bills via mobile messages and pay through mobile banking. For groups sharing a water connection, one designated member collects a share of the payment from each user and pays the total amount to WASA. Monthly bills typically vary based on the meter readings, ranging from BDT80 to 150 (around £1 or less) per user.

Site A’s mission for an accessible legal water supply continues with new installations and improvements to the CBO network. The network of CBOs also advocates for subsidized water bills for low-income communities, resulting in a revised billing system in 2022, where residents of informal settlements pay a lower rate for water. The current rate for these residents is BDT13 (£0.079) per 1000 L.

The legalization of water connections in Site A exemplifies community solidarity and resilience and effective grassroots advocacy. The collaboration between residents and NGOs shows how partnerships can drive social change and empower communities through the establishment of the CBO as a liaison with Dhaka WASA. However, several critical issues persist, such as the accountability of CBO leaders in managing water supply processes and the disparities in deposits and installation costs among residents. Local elites and political figures opposed formalization, fearing loss of control and revenue from informal arrangements.

Local political leaders did not support our initiatives… sometimes they even blocked legal connections to protect their own interests. (Male Community Leader, Site A, GD)

Despite these obstacles, the CBOs leveraged both bottom-up community pressure and top-down negotiation to assert residents’ right to safe water. The introduction of a subsidized billing system in 2022 is a positive step, but the sustainability of these efforts relies on ongoing advocacy and support from NGOs and government agencies. Overall, Site A’s experience reflects both the successes and challenges marginalized communities faced in accessing essential public services. This initiative highlights community-led efforts to achieve justice in health access and long-term sustainability through institutional reform. While significant progress has been made, continuous engagement and structural reform are necessary to ensure equitable access to water for all urban residents.

This initiative reflected key aspects of hybrid governance: the collaboration of formal actors (WASA, city officials), informal leaders (CBOs, local influencers), and NGOs, each playing complementary roles. NGOs played a critical bridging role, facilitating advocacy, infrastructure development, and dialogue with state actors.

#### Example 2: Collective actions for mobilizing emergency treatment funds

Research participants from both sites mentioned a common internal hybrid arrangement of emergency resource mobilization to financially support poor patients’ treatment. Participants recalled incidents where emergency funds were mobilized through donations from residents, community leaders, and local shopkeepers/businesses to support poor residents requiring emergency medical support (such as childbirth and injuries). Sometimes, community leaders or influential people took the lead to mobilize emergency funds, either doing this themselves or assigning their associates to this task. Sometimes, house owners or their managers (informally appointed among the tenants with a good relationship with the house owners) collected small donations from other tenants. Participants shared that residents willingly contribute to these emergency medical funds according to their financial capacity (e.g. someone would donate £0.60, someone £1 or more). One male local elite from Site B stated:

Suppose one poor person suddenly falls sick and needs to be immediately hospitalized. But that family has no money, even for transportation, to take him/her to the hospital. In that emergency situation, we request their neighbourhood to donate as much as possible. Some people give BDT 10, 20, or 50 [between £0.06 and £0.31], or whatever they can afford. Sometimes, I assign 2 or 3 people I know to collect money from the neighborhood. Then we rent vehicles and take the sick person to the public hospitals according to the types/severity of the health conditions. (Male, Secretary of an NGO-formed committee, Site B, GNM)

However, not all residents benefited from this community-led emergency fund mobilization. Long-term residents who built strong social relationships and had political networks were more likely to receive financial help during medical emergencies. New migrants or families without connections reported being excluded from these community initiatives, as illustrated in the following quote from a temporary factory worker who lived in Site B for 7 years but had limited social and no political connections. As the sole earner in her family, she struggled to manage financially and needed money for her husband’s treatment.

Doctors prescribed my husband a CT scan, which cost BDT4000 (£24). I could not manage the money with my limited income. Since I do not know anyone here, I have no one to turn to for help. I initially reached out to some community leaders for help. Some said they would help, but never did. So, I gradually stopped asking for help. (Female Factory Worker, Site B, GD)

In Site A, local young people (mostly youth club members) often took the initiative to mobilize emergency treatment funds for poor people. According to them, when a poor person needs emergency medical support, they first discuss this need with the “senior brothers” (local youth leaders) and then work together to collect donations from the community. The funds raised were then used to help sick or injured residents receive treatment. We observed young people in Site A proactively and voluntarily taking initiatives to help residents during a fire incident that happened during data collection. They actively participated in rescuing people, took them to hospitals, and mobilized treatment funds for those who could not afford it, regardless of social status and political differences. It might be possible that these youth-led initiatives are politically motivated—looking after community welfare for political support.

The practice of emergency fund mobilization in Sites A and B reflects a vital, though uneven, form of hybrid governance, where informal actors compensate for the absence of state-provided safety nets. In contexts of systemic exclusion, these initiatives highlight the communities’ resilience and collective care. However, they also expose deep-rooted inequalities in local power ecologies, where support depends on one’s embeddedness within existing networks and is shaped by social proximity, visibility, and perceived loyalty. While the collective mobilization of resources demonstrates social solidarity, it also reveals how hybrid governance can reproduce selective exclusion, where support is discretionary, and assistance may be tied to long-term social and political capital. These dynamics illustrate that even the most humanitarian practices in informal settlements are embedded in the broader architecture of negotiated power and informal governance.

#### Example 3: Hybrid governance arrangement for patient referrals

The community health workers (CHWs) of an NGO (here referred to as NGO X) in Site B established a referral mechanism through a hybrid governance arrangement. Initially, NGO X CHWs observed the low uptake of child immunization and maternal healthcare services and identified financial constraints as the key barrier. Although poor residents of Site B could access these services free of charge from public hospitals, these hospitals were 8–20 km from Site B. As a consequence, Site B residents visited those hospitals only for perceived severe health conditions/diseases.

Another NGO operated a clinic (here referred to as NGO Y clinic) near Site B, which provided maternal and child health services at a subsidized cost to poor people.

Although this clinic charged only a minimum fee for child immunization and maternal healthcare, the extremely poor people from Site B could not afford it. NGO X CHWs informed their supervisors of this barrier, which was eventually communicated to the headquarters of NGO X. Then, as a “service integration” strategy, NGO X arranged several coordination meetings with NGO Y officials and requested reduced fees and free services for child immunization and maternal healthcare for extremely poor people. After several coordination meetings and lots of informal negotiations at the higher level, both NGOs’ senior management informally agreed on establishing a referral system considering it mutually beneficial for the performance of both NGOs. NGO X CHWs would refer Site B’s poor people with a handwritten note, termed “referral slips”, to the NGO Y clinic for maternal and child healthcare. NGO X CHWs assessed the financial status of Site B residents, following their organizational policy and mentioned the amount of subsidy required for each resident on the relevant “referral slip”, based on the residents’ financial capacity to pay for services. One NGO X CHW explained:

On the referral slips, we wrote the name and age of service recipients and the service s/he will receive and then signed at the bottom of the slips. We also mentioned the amount of money they could pay. For example, if the service charge was BDT100 (£0.62), this patient would pay BDT50 (£0.32), or that patient would pay BDT30 (£0.19). We recommended 50 taka for those in the P category (indicated to “Poor”) and 30 or 20 taka or “free” for those in the UP category (indicated to “Ultra Poor”). They (the NGO Y clinic) charged our referred patients accordingly. (Female, NGO CHW, Site B, GNM)

NGOs and community members benefited from this hybrid arrangement of referral system, where NGO X’s CHWs acted as community mobilizers for NGO Y’s clinic by referring Site B residents. This resulted in increased patient flow to the clinic and contributed to its performance achievements, which motivated NGO Y to participate in this arrangement. Additionally, the acceptance and social recognition of the NGO X CHWs in Site B increased as they helped people access low-cost or free services.

Even after NGO X discontinued their health services in Site B, the CHWs of both NGOs informally continued referring patients, but through verbal communications over the phone without referral slips.

The community members do not realize that we are no longer working as CHWs since our NGO has ceased its activities in this area. Previously, they would approach us to request reduced fees at the NGO Y clinic. I advise them to visit the clinic and connect me with the nurse or clinic staff via mobile phone. When I communicate with the clinic staff, they are willing to reduce the service charges. The clinic staff still cooperate with us because we are unofficially helping with community mobilization for them, which allows them to attract more patients and improve their performance. If we do not refer people to their clinic, they will not receive enough patients. (Former Female CHW, NGO X, Site B, GNM)

This situation exemplifies a hybrid governance arrangement because the referral system combined both formal and informal elements. Initially, the arrangement started informally, became more formal over time, and then shifted back to include more informal components. The initiative began with NGO X CHWs informally identifying a problem and seeking a solution. The higher authorities of both NGOs initiated formal negotiations and agreed on an informal arrangement without formal documentation. The system operated on trust, relying on the financial assessment by the NGO X CHWs and the expectation that they would not abuse the system. On the formal side, the arrangement included a strategic agreement by the authorities at a higher level, as well as record-keeping through referral slips. It was recognized as a legitimate process by all parties involved.

### Drivers and challenges of hybrid governance

While this MHG system offers promising avenues for enhancing urban health systems, it also poses challenges related to coordination, compatibility of governance structures, and potential conflicts among actors. By capturing learnings from the above examples and analyzing them using the MHG framework, we identified some interchangeable drivers and challenges of hybrid governance arrangements in informal urban settlements (Table 2). Experiential knowledge was captured and translated using the MHG framework, offering valuable insights for learning about health systems in urban contexts.

**Table 2.** Key drivers and challenges of hybrid governance in informal urban settlements

Themes	Key drivers	Key challenges
Governance layers and actor positionality	Diverse actors (state, NGOs, community leaders) bring complementary strengths Flexibility in roles and actor positioning enhances responsiveness	Power imbalances due to patronage, nepotism, and elite control Politically influenced leadership reinforce inequity
Interactions and relationships	Personal relationships and informal negotiations facilitate collaboration Mutual benefits drive alliances	Lack of formal accountability among local governance actors Service access dependent on loyalty and connections, not rights
Shared social space and networked governance	Collaborations and partnerships between formal and informal actors create adaptive, context-specific, and resilient governance Informal norms guide cooperation in the absence of formal rules	Absence of clear governance structures produces ambiguity and conflict Transactional cooperation undermines long-term sustainability
Hardware, software, and trust	Synergy between physical resources (hardware) and social capital (software) enables service delivery Instrumental trust promotes short-term coordination	Low trust in governance actors and formal health systems challenge sustainability Dependence on material incentives hinders institutionalization
Examples of practice	Local fundraising and emergency response mechanisms show community resilience and solidarity Informal referral systems link community needs to formal services	

Hybrid governance in informal urban settlements emerges as both a necessary workaround and a pragmatic adaptation in contexts where formal state infrastructure is weak or absent (Boege et al. 2009b). The MHG framework, as described in the Bangladesh context, demonstrates how governance in informal settings is negotiated and relational.

The examples presented here also exemplify hybrid governance, as defined by Boege et al. (2009a), as governance in zones of limited statehood where non-state actors fill governance gaps. As presented in the above three examples, flexible roles of actors and the capacity of informal arrangements to mobilize resources and deliver services are the key drivers of this hybrid system. Examples 1 and 3 illustrate how NGOs acted as intermediaries by leveraging their institutional capacity and informal alliances to facilitate health service access for residents. These examples also highlight this system’s potential for innovation and responsiveness.

Trust in hybrid governance is instrumental (Gilson 2003), where actors cooperate based on strategic benefits and legitimate rights, such as access to water in Example 1. The MHG framework also illustrates the crucial importance of “software” governance (relationships, norms, collaboration, and motivation) (Sheikh et al. 2011) as important as infrastructure or finance.

However, challenges are equally prominent. These hybrid systems operate as “shadow systems” (Cleaver 2002), which are essential yet often fragile, revealing a paradox where informality provides both a solution and a barrier to justice and sustainable governance for health service provision in informal settlements. This type of governance system is vulnerable to political shifts, fluid power structures, and institutional discontinuity, which limits its long-term viability (Ostrom 1990). Heavy reliance on patron–client relationships in hybrid governance reinforces inequality (Banks et al. 2020). Hossain (2013) critiqued this phenomenon as “informal politics”, where marginalized populations are served but not empowered. Furthermore, accountability of informal actors is a significant challenge, which can lead to inequitable or arbitrary service provision (Freire and Stren 2001). Sustainability of hybrid governance arrangement depends on individual actors and informal agreements, leaving the system vulnerable to actor exit or national and local political change.

We observe some site-specific unique characteristics of hybrid governance in our study sites. For example, the long history of NGO presence in Site A contributed to the leadership development and political consciousness of Site A residents, which is reflected in the legalization of supply water connections (Example 1) and the resource mobilization by youth leaders (Example 2). In contrast, community collective actions in Site B depended on local elites (Example 2) and access to affordable NGO health services through CHWs (Example 3).

## Conclusion

This paper explores the complex hybrid governance systems of public health service delivery and access in Dhaka’s informal urban settlements. The MHG framework introduced in this paper highlights both the adaptability and resilience and the fragility and inequity of these systems. This paper presents hybrid governance as a resilient-oriented governance system, offering an alternate pathway to negotiate justice. These findings also contribute to learning about health systems in fragile urban settings, such as informal settlements. This paper advocates for a nuanced understanding of local governance systems and community resilience, which are crucial for establishing a resilient governance system that effectively delivers health services in complex urban environments. Policymakers, city authorities, and service providers must recognize the realities of hybrid governance to enhance public health service delivery in informal urban settlements. This includes integrating informal networks that these communities rely on in planning and implementation processes to create a more inclusive health service delivery model.

Overall, the MHG framework effectively captures the complexities of interactions among diverse actors, highlights the critical role of informality in public service delivery, and emphasizes governance’s embeddedness within intricate local power dynamics. By viewing hybrid governance as a negotiated, relational, and adaptive system, the MHG framework provides a valuable analytical tool for researchers and policymakers to navigate the multifaceted governance challenges of urban health systems.

In conclusion, hybrid governance systems are a crucial, context-sensitive mechanism for health service delivery in informal settlements. Nonetheless, their effectiveness is limited by underlying power imbalances, instrumental trust, and a lack of institutionalization. Policy responses should recognize these complexities, leverage the strengths of hybrid governance, and address its structural weaknesses.

## Data Availability

The data underlying this article cannot be shared publicly to safeguard the privacy and identity of the research participants because the information gathered in this research is locally politically sensitive. Anonymized data can be shared on reasonable request to the corresponding author.

## Reflexivity statement

The research team consists of both early-career and experienced public health researchers from Bangladesh and the UK. This paper is based on data from the PhD project of the lead author, B.A. The other four authors, K.O., S.F.R., L.W., and S.T., served as her PhD supervisors. As citizens of Bangladesh and residents of Dhaka city, B.A. and S.F.R. have a deeper understanding of the city’s sociopolitical dynamics. Their extensive work experience in urban settings and informal urban settlements in Bangladesh has further enriched their understanding of community dynamics, local power structure and politics, and the complexities surrounding access to health services in these contexts. K.O., a UK-based researcher with expertise in community-based participatory research approaches (CBPR), guided B.A. to learn and apply CBPR principles throughout the research process, which is reflected in this paper. S.T. and L.W., two experienced UK-based health systems researchers with extensive research experience in the Bangladesh context, contributed a global perspective and intersectional analysis to the discussion. Therefore, the authors provide both “insider” and “outsider” perspectives. However, their differing socioeconomic status distances them from the communities in informal settlements. Engaging community researchers throughout the research process enabled academic researchers to gain valuable “insider” insights. As community representatives with deep understandings of their communities, they provided in-depth knowledge about local governance dynamics, which other methods may overlook due to the sensitive nature of the topics being explored. Academic researchers and community researchers mutually support each other by exchanging knowledge, which has enhanced data analysis and interpretation.

## References

[czaf081-B1] Abdulhadi R, Bailey A, Van Noorloos F. Access inequalities to WASH and housing in slums in low- and middle-income countries (LMICs): a scoping review. Glob Public Health 2024;19:2369099. 10.1080/17441692.2024.2369099 38940272

[czaf081-B2] Adams AM, Islam R, Ahmed T. Who serves the urban poor? A geospatial and descriptive analysis of health services in slum settlements in Dhaka, Bangladesh. Health Policy Plan 2015;30:i32–45. 10.1093/heapol/czu094 25759453 PMC4353891

[czaf081-B3] Afsana K, Wahid SS. Health care for poor people in the urban slums of Bangladesh. Lancet 2013;382:2049–51. 10.1016/S0140-6736(13)62295-3 24268606

[czaf081-B4] Anciano F, Lombard M. Hybridising governance for resilience in a time of crisis: learning from community-based organisations in Cape Town and Cali. Int Dev Plan Rev 2024;47:41–63. 10.3828/idpr.2024.15

[czaf081-B5] Arise Consortium . Improving accountability for equitable health and well-being in urban informal spaces: moving from dominant to transformative approaches. Prog Dev Stud 2024;24:301–20. 10.1177/14649934231225530

[czaf081-B6] Banks N . A tale of two wards: political participation and the urban poor in Dhaka city. Environ Urbaniz 2008;20:361–76. 10.1177/0956247808096116

[czaf081-B7] Banks N, Lombard M, Mitlin D. Urban informality as a site of critical analysis. J Dev Stud 2020;56:223–38. 10.1080/00220388.2019.1577384

[czaf081-B8] Banks N, Roy M, Hulme D. Neglecting the urban poor in Bangladesh: research, policy and action in the context of climate change. Environ Urbaniz 2011;23:487–502. 10.1177/0956247811417794

[czaf081-B9] BBS . *Population and Housing Census 2022: National Report* (Volume I). Dhaka, Bangladesh. 2023.

[czaf081-B10] Bingham AJ . From data management to actionable findings: a five-phase process of qualitative data analysis. Int J Qual Methods 2023;22:1–11. 10.1177/16094069231183620

[czaf081-B11] Boege V, Brown A, Clements K et al Building peace and political community in hybrid political orders. Int Peacekeep 2009a;16:599–615. 10.1080/13533310903303248

[czaf081-B12] Boege V, Brown MA, Clements KP. Hybrid political orders, not fragile states. Peace Rev 2009b;21:13–21. 10.1080/10402650802689997

[czaf081-B13] Brondizio ES, Ostrom E, Young OR. Connectivity and the governance of multilevel social-ecological systems: the role of social capital. Annu Rev Environ Resour 2009;34:253–78. 10.1146/annurev.environ.020708.100707

[czaf081-B14] Cleaver F . Reinventing institutions: bricolage and the social embeddedness of natural resource management. Eur J Dev Res 2002;14:11–30. 10.1080/714000425

[czaf081-B15] Corburn J, Sverdlik A. Slum upgrading and health equity. Int J Environ Res Public Health 2017;14:342. 10.3390/ijerph14040342 28338613 PMC5409543

[czaf081-B16] Cornea NL . *Urban Political Ecology*, *in Oxford Bibliographies*. Oxford University Press, 2019. 10.1093/OBO/9780199874002-0203

[czaf081-B17] Cornea NL, Véron R, Zimmer A. Everyday governance and urban environments: towards a more interdisciplinary urban political ecology. Geogr Compass 2017;11:e12310. 10.1111/gec3.12310

[czaf081-B18] Creswell JW, Creswell JD. *Research Design: Qualitative, Quantitative and Mixed Methods Approaches*, 5th edn. Los Angeles, USA: SAGE Publications, Inc, 2018. https://spada.uns.ac.id/pluginfile.php/510378/mod_resource/content/1/creswell.pdf

[czaf081-B19] Devas N, Amis P, Beall J et al *Urban Governance, Voice and Poverty in the Developing World*. London: Earthscan Publications Ltd, 2004. 10.4324/9781849773683

[czaf081-B20] Elsey H, Agyepong I, Huque R et al Rethinking health systems in the context of urbanisation: challenges from four rapidly urbanising low-income and middle-income countries. BMJ Glob Health 2019;4:e001501. 10.1136/bmjgh-2019-001501 PMC6577312 31297245

[czaf081-B21] Ezeh A, Oyebode O, Satterthwaite D et al The history, geography, and sociology of slums and the health problems of people who live in slums. Lancet 2017;389:547–58. 10.1016/S0140-6736(16)31650-6 27760703

[czaf081-B22] Freire M . The Challenges of Urban Government: Introduction. In: Freire M, Stren RE (eds.), *The Challenge of Urban Government: Policies and Practices*. The World Bank Institute, Washington, D.C. The Centre for Urban and Community Studies, University of Toronto: WBI Development Studies, 2001, xvii–xli.

[czaf081-B23] Gilson L . Trust and the development of health care as a social institution. Soc Sci Med 2003;56:1453–68. 10.1016/S0277-9536(02)00142-9 12614697

[czaf081-B24] Goldsmith LJ. Using framework analysis in applied qualitative research. Qualitative Report 2021;26(6):2061–2076. 10.46743/2160-3715/2021.5011

[czaf081-B25] Goodfellow T . Political informality: deals, trust networks, and the negotiation of value in the urban realm. J Dev Stud 2020;56:278–94. 10.1080/00220388.2019.1577385

[czaf081-B26] Hackenbroch K, Hossain S. The organised encroachment of the powerful”-everyday practices of public space and water supply in Dhaka, Bangladesh. Plan Theory Pract 2012;13:397–420. 10.1080/14649357.2012.694265

[czaf081-B27] Heynen N, Kaika M, Swyngedouw E. Urban political ecology—politicizing the production of urban natures. In: Heynen N, Kaika M, Swyngedouw E (eds.), *In the Nature of Cities—Urban Political Ecology and the Politics of Urban Metabolism*, 1st edn. London: Routledge, 2006, 16–35. https://www.taylorfrancis.com/books/9781134206476/chapters/10.4324/9780203027523-8

[czaf081-B28] Hossain S . The production of space in the negotiation of water and electricity supply in a Bosti of Dhaka. Habitat Int 2012;36:68–77. 10.1016/j.habitatint.2011.05.007

[czaf081-B29] Hossain S . The informal practice of appropriation and social control—experience from a Bosti in Dhaka. Environ Urbaniz 2013;25:209–24. 10.1177/0956247812465803

[czaf081-B30] Jones P . Formalizing the informal: understanding the position of informal settlements and slums in sustainable urbanization policies and strategies in Bandung, Indonesia. Sustainability 2017;9:1436. 10.3390/su9081436

[czaf081-B31] Kaur M, Sharma A, Vijin PP et al Exploring the complexities of slum vulnerability in Haryana, India: a qualitative research journey into economic, social, physical, and health dimensions. Int J Qual Stud Health Well-Being 2025;20:2432692. 10.1080/17482631.2024.2432692 39676514 PMC11650438

[czaf081-B32] Kickbusch I, Gleicher D. *Governance for Health in The 21st century*. Copenhagen, Denmark: WHO Regional Office for Europe, 2012, 1–106.

[czaf081-B33] Lemos MC, Agrawal A. Environmental governance. Annu Rev Environ Resour 2006;31:297–325. 10.1146/annurev.energy.31.042605.135621

[czaf081-B34] Loureiro M, Joshi A, Barnes K et al *IDS Working Paper 534—Governance Diaries: An Approach to Governance Research from the Ground Up*, Vol. 2020. Brighton, UK: The Institute of Development Studies and Partner Organisations, 2020. https://hdl.handle.net/20.500.12413/15119

[czaf081-B35] March H, Swyngedouw E. Chapter 1: resilience for all or for some? Reflections through the Lens of urban political ecology. In: The Urban Book Series. Cham: Springer International Publishing, 2022, 3–19.

[czaf081-B36] McCann E . Governing urbanism: urban governance studies 1.0, 2.0 and beyond. Urban Stud 2017;54:312–26. 10.1177/0042098016670046

[czaf081-B37] Mcdermott AM, Hamel LM, Steel D et al Hybrid healthcare governance for improvement? Combining top-down and bottom-up approaches to public sector regulation. Public Adm 2015;93:324–44. 10.1111/padm.12118

[czaf081-B38] Meagher K . Informal Economies and Urban Governance in Nigeria: Popular Empowerment or Political Exclusion? African Studies Review 2011;54:47–72. 10.1353/arw.2011.0026

[czaf081-B39] Ostrom E . *Governing the Commons: the Evolution of Institutions for Collective Action*, 1st edn. Cambridge, UK: Cambridge University Press, 1990. 10.1017/CBO9780511807763

[czaf081-B40] Paller JW . Informal networks and access to power to obtain housing in urban slums in Ghana. Afr Today 2015;62:31–55. 10.2979/africatoday.62.1.31

[czaf081-B41] Parizeau K . Urban political ecologies of informal recyclers’ health in Buenos Aires, Argentina. Health Place 2015;33:67–74. 10.1016/j.healthplace.2015.02.007 25770437

[czaf081-B42] Rice JL . An urban political ecology of climate change governance. Geogr Compass 2014;8:381–94. 10.1111/gec3.12134

[czaf081-B43] Roy A . Urban informality: toward an epistemology of planning. J Am Plann Assoc 2005;71:147–58. 10.1080/01944360508976689

[czaf081-B44] Schiffer E . *Manual - Net-Map Toolbox: Influence Mapping of Social Networks*. International Food Policy Research Institute, 2007. https://netmap.wordpress.com/wp-content/uploads/2008/06/net-map-manual-long1.pdf

[czaf081-B46] Sheikh K, Abimbola S. *Learning Health Systems: Pathways to Progress*, Flagship Report of the Alliance for Health Policy and Systems Research (Geneva, 2021). https://www.ars.ahpsr.org/learning-systems-flagship-report (23 May 2025, date last accessed)

[czaf081-B47] Sheikh K, Gilson L, Agyepong IA et al Building the field of health policy and systems research: framing the questions. PLoS Med 2011;8:e1001073. 10.1371/journal.pmed.1001073 21857809 PMC3156683

[czaf081-B48] Swyngedouw E . Governance innovation and the citizen: the Janus face of governance-beyond-the-state. Urban Stud 2005;42:1991–2006. 10.1080/00420980500279869

[czaf081-B49] Swyngedouw E, Heynen NC. Urban political ecology, justice and the politics of scale. Antipode 2003;35:898–918. 10.1111/j.1467-8330.2003.00364.x

[czaf081-B50] te Lintelo DJH, Gupte J, McGregor JA et al Wellbeing and urban governance: who fails, survives or thrives in informal settlements in Bangladeshi cities? Cities 2018;72:391–402. 10.1016/j.cities.2017.10.002

[czaf081-B51] The Daily Star . Cross-Sector Collaboration Key to Health Reforms, *The Daily Star*, 13 May 2025. https://www.thedailystar.net/news/bangladesh/news/cross-sector-collaboration-key-health-reforms-3893246?utm_source=chatgpt.com (23 May 2025, date last accessed)

[czaf081-B52] The New Age . Early Execution of Health Sector Reforms Warranted. *The New Age*, 22 May 2025. https://www.newagebd.net/post/editorial/265485/early-execution-of-health-sector-reforms-warranted?utm_source=chatgpt.com (23 May 2025, date last accessed)

[czaf081-B53] The Prothom Alo . Reform Commission Recommends Allocating 15pc of National Budget to Health Sector. *The Prothom Alo*, 6 May 2025. https://en.prothomalo.com/bangladesh/x26rkpa76h (23 May 2025, date last accessed)

[czaf081-B54] UN-Habitat . World Cities Report 2022, 2022. https://unhabitat.org/wcr/ (6 August 2023, date last accessed)

[czaf081-B55] World Bank . *Bangladesh - Dhaka: Improving Living Conditions for the Urban Poor, 2007*, Vol. 1, 35824. Washington, DC: The World Bank, 2007. http://documents.worldbank.org/curated/en/587231468007834055

[czaf081-B56] Yasmin T, Farrelly MA, Rogers BC et al Hybrid and multi-level adaptive governance for sustainable urban transformations in the global south: a secondary city case study. Front Water 2022;4:756273. 10.3389/frwa.2022.756273

[czaf081-B57] Yiftachel O . Critical theory and “gray space”: mobilization of the colonized. City 2009;13:246–63. 10.1080/13604810902982227

